# Integrative Investigation of Lactylome–Proteome Interplay in Diabetic Cardiomyopathy for Pinpointing Disease Development–Associated Pathways or Proteins

**DOI:** 10.1155/jdr/3543697

**Published:** 2026-04-24

**Authors:** Di Ma, Wenjie Cai, Hui Yuan, Meixin Shi, Ye Jin, Yifeng Cui, Peng Liu, Xi Liu, Can Wei

**Affiliations:** ^1^ Department of Pathophysiology, School of Basic Medical Sciences, Harbin Medical University, Harbin, Heilongjiang, China, hrbmu.edu.cn; ^2^ School of Basic Medical Sciences, Mudanjiang Medical University, Mudanjiang, Heilongjiang, China, mdjmu.cn; ^3^ Department of General Surgery, Key Laboratory of Hepatosplenic Surgery, Ministry of Education, The First Affiliated Hospital of Harbin Medical University, Harbin, Heilongjiang, China, hrbmu.edu.cn; ^4^ Department of Cardiology, Ordos Central Hospital, Ordos, Inner Mongolia Autonomous Region, China

**Keywords:** diabetic cardiomyopathy, lactylome, protein lactylation, proteome

## Abstract

**Background and Aim:**

Lysine lactylation (Kla) has emerged as a novel posttranslational modification implicated in various disease processes, yet its role in diabetic cardiomyopathy (DCM) pathogenesis remains unknown. The objective of this study was to ascertain whether protein lactylation is involved in DCM progression.

**Methods:**

Proteomic and lactate analysis via liquid chromatography with tandem mass spectrometry was performed on the heart tissues of db/m mice (as the control group) and db/db mice (as the DCM group). Subsequently, a series of bioinformatics analyses was employed to analyze the Kla site and Kla‐modified proteins in the two groups.

**Results:**

Bioinformatics analysis revealed a greater abundance of Kla sites in the DCM group than in the control group. In addition, subcellular localization analysis indicated that Kla‐modified proteins were predominantly located in the cytoplasm and mitochondria. Protein lactylation modification mainly occurred on histone H2, and in comparison to the control group, modification of the H4C1‐K32 site was notably elevated in the DCM group. Furthermore, 113 significantly modified Kla sites were associated with 78 modified proteins in the DCM group, whereas 37 significantly modified Kla sites were associated with 25 modified proteins in the control group. These Kla‐modified proteins participated in biological processes and pathways related to glucose metabolism and DCM. Finally, five candidate sites were identified using random forest, LASSO regression, support vector machine–recursive feature elimination, and logistic regression: A2ASS6_K928_Ttn, A2ASS6_K13499_Ttn, Q61425_K212_Hadh, Q8K2B3_K517_Sdha, and Q9R0Y5_K100_Ak1.

**Conclusions:**

Our findings suggest that protein lactate modification in the lactylome and proteome could be a promising treatment for DCM. This provides a reliable basis for further investigating the roles of Kla and Kla‐modified proteins to develop new and effective therapeutic targets for treating DCM.

## 1. Introduction

Diabetic cardiomyopathy (DCM), one of the most serious cardiovascular complications of diabetes and a major cause of death in diabetic patients [1], is marked by myocardial dysfunction caused by chronic hyperglycemia [2]. The main pathological features consist of myocardial hypertrophy, myocardial fibrosis, and compromised ventricular function. These manifestations have the potential to trigger extensive myocardial necrosis. As the prevalence of diabetes increases each year, the prevalence of DCM also increases [3]. Despite increasing awareness of DCM and the emergence of new treatment methods, there is a lack of effective treatment for DCM in clinical practice [4]. Given the current lack of diagnostic criteria and therapeutic targets for DCM, elucidating the pathogenesis of DCM, uncovering diagnostic biomarkers, and formulating therapeutic targets hold substantial clinical importance.

Researchers commonly explore the fundamental mechanisms of DCM in terms of insulin resistance, excessive oxidative stress, inflammatory response, and mitochondrial dysfunction. However, given the complex factors contributing to these unregulated and persistent pathological alterations, it remains challenging to comprehensively clarify these mechanisms. With the development of sequencing technology, increasingly more epigenetic regulation mechanisms have been studied [5–6]. Epigenetics pertains to inheritable, reversible patterns in genetic expression that do not alter the DNA sequence and are intricately associated with environmental factors [7–8]. DCM is often affected by environmental elements like glycolipid homeostasis, which can have a substantial impact on the epigenetic state. Thus, epigenetic regulation may play a crucial role in DCM pathogenesis. Emerging studies have discovered correlations between DCM and multiple epigenetic regulatory mechanisms, including DNA methylation, histone alterations, and other epigenetic components [9].

Posttranslational modification of proteins is a key component of epigenetics and governs numerous biological and pathological processes in humans [10–11]. The main types of posttranslational modification of proteins comprise methylation, acetylation, glycosylation, phosphorylation, and ubiquitination of amino acid residues [12–13]. Until 2019, mass spectrometry was the primary technique used to detect protein lactylation, a novel contributor to posttranslational modification [14]. In recent years, increasing evidence has suggested that lactate functions not only as a metabolic product but also as a critical signaling molecule regulating cellular processes through protein lactylation [15–16]. Accumulating studies have demonstrated that lactylation participates in diverse biological processes, including neurodevelopment, metabolic regulation, tumors, and inflammation [17–19]. These findings highlighted lactylation as a potential molecular process connecting metabolic dysregulation and disease progression. Considering that DCM depends on aerobic glycolysis to generate lactic acid, the identification of protein lactylation provides a novel vantage point for delving into the pathogenesis of DCM.

To the best of our knowledge, the characteristics of protein lactylation modification in DCM have not been reported previously. This study analyzed the heart tissues of control and DCM mice via an integrated examination of the lactylome and proteome and obtained a complete protein lactate modification map. The aim was to develop a method for discovering DCM‐associated protein lactylation modification targets.

## 2. Material and Methods

### 2.1. Animals and Tissue Samples

The DCM model was established using homozygous leptin receptor‐lacking (db/db) mice, whereas heterozygous (db/m) mice (obtained from Harbin Medical University) served as the control group. All mice were reared on a regular diet in an environment with standard humidity and temperature conditions. For a duration of 24 weeks, mice in both groups were fed a regular diet. On the last day, the mice in the control group (*n* = 4 per group) and DCM group (*n* = 4 per group) were sacrificed using CO_2_ anesthesia, and heart tissues were removed under sterile conditions for further detection. All mice were handled in accordance with *The Guide for the Care and Use of Laboratory Animals,* adopted and announced by the United States NIH. Every treatment plan was sanctioned by the Institutional Animal Care and Use Committee at the Experimental Animal Center of Harbin Medical University (located in Harbin, Heilongjiang Province, China).

### 2.2. Protein Extraction

The sample was pulverized into cell powder using liquid nitrogen, and then transferred to a 5‐mL centrifuge tube. Subsequently, four times the volume of lysis buffer (comprising 1% Triton X‐100 and 1% protease inhibitor cocktail) was added to the cell powder. Next, the mixture was sonicated for 3 min on ice using a high‐intensity ultrasonic processor (Scientz). The remaining debris was separated by centrifugation at 12,000 g for 10 min at 4°C. Ultimately, the supernatant was gathered, and the protein concentration was measured using a bicinchoninic Acid (BCA) kit following the manufacturer′s guidelines.

### 2.3. Trypsin Digestion

The protein sample was slowly added to a trichloroacetic acid (TCA) stock solution to achieve a final 20% (m/v) TCA concentration, enabling protein precipitation. Then, the mixture was vortexed until mixed thoroughly, incubated at 4°C for 2 h, and centrifuged at 4500 g for 5 min at 4°C to collect the precipitate. The precipitated protein was washed three times with precooled acetone and air‐dried for 1 min. Next, the protein sample was redissolved in 200‐mM triethylammonium bicarbonate solution and dispersed through sonication. Trypsin was added at a trypsin‐to‐protein mass ratio of 1:50 for initial overnight digestion. The sample was reduced with 5‐mM dithiothreitol at 56°C for 30 min, then alkylated with 11‐mM iodoacetamide in the dark at room temperature for 15 min. Finally, the peptides were desalted using a Strata X SPE column.

### 2.4. Affinity Enrichment

For the enrichment of modified peptides, tryptic peptides dissolved in NETN buffer (composed of 100 mM NaCl, 1 mM EDTA, 50 mM Tris‐HCl, 0.5% NP‐40, with a pH of 8.0) were incubated with prewashed antibody beads at 4°C overnight, accompanied by gentle agitation. Subsequently, the beads were washed four times with NETN buffer and twice with water. The bound peptides were eluted from the beads using 0.1% trifluoroacetic acid. Eventually, the eluted fractions were pooled together and dried under vacuum. Regarding liquid chromatography with tandem mass spectrometry (LC‐MS/MS) analysis, the resultant peptides were desalted with C18 ZipTips (Millipore), following the manufacturer′s instructions.

### 2.5. Proteome and Lactylome Analyses via LC‐MS/MS

The tryptic peptides were dissolved in Solvent A and directly loaded onto a self‐made reversed‐phase analytical column (25 cm in length, 100 *μ*m in inner diameter). The mobile phase was composed of Solvent A (0.1% formic acid, 2% acetonitrile in water) and Solvent B (0.1% formic acid in acetonitrile). Following proteome analysis, the peptides were separated using the following gradient: from 0 to 5 min, the proportion of Solvent B increased from 9% to 24%; from 5 to 6 min, it rose from 24% to 35%; from 6 to 7 min, it increased from 35% to 80%; and from 7 to 8 min, it remained at 80%. These separations were carried out at a constant flow rate of 600 nL/min on a NanoElute UHPLC system (Bruker Daltonics).

For the lactylome analysis, peptides were separated using the following gradient: from 0 to 14 min, Solvent B increased from 6% to 22%; from 14 to 16 min, it rose from 22% to 30%; from 16 to 17 min, it increased from 30% to 80%; and from 17 to 20 min, it remained at 80%. All of this was done at a constant flow rate of 450 nL/min on a NanoElute UHPLC system (Bruker Daltonics). The peptides were then processed through a capillary source and analyzed by timsTOF HT mass spectrometry. An electrospray voltage of 1.6 kV was applied. Both precursors and fragments were examined by the TOF detector. The timsTOF Pro was operated in the data‐independent parallel accumulation serial fragmentation (dia‐PASEF) mode.

For the proteome analysis, the full MS scan was set within the range of 300–1500 (MS/MS scan range), and 21 PASEF MS/MS scans were obtained per cycle. The MS/MS scan range was set from 380 to 1170, and the isolation window was set at 10 m/z. For the lactylome analysis, the full MS scan was set within the range of 100–1700 (MS/MS scan range), and eight PASEF (MS/MS mode)–MS/MS scans were obtained per cycle. The MS/MS scan range was set within the range of 425–1025, and the isolation window was set at 25 m/z.

### 2.6. Database Search

Data were processed using Spectronaut (v.18) software. Tandem mass spectra were searched against Mus_musculus_10090_SP_20231220.fasta (comprising 17,191 entries), which was concatenated with a reverse decoy database. For the proteome analysis, trypsin/P was designated as the cleavage enzyme, with the allowance of a maximum of two missed cleavages, whereas for the lactylome analysis, up to four deletions for cleavage were allowed. Carbamidomethylation of cysteine was defined as a fixed modification. N‐terminal acetylation of the protein and methionine oxidation were defined as variable modifications. The false discovery rates were set to be less than 1% for peptide‐spectrum matches, site identifications, and protein identifications. Protein group quantification based on intensity was retrieved from the MaxQuant result files to signify the expression of a specific protein among different samples. The Kla‐peptide levels were computed according to the raw spectral intensity. To offset changes in Kla‐peptide levels resulting from protein level fluctuations, the abundance of each identified Kla‐peptide was normalized using the corresponding protein abundance.

### 2.7. Bioinformatics Analysis

The quantitative data of Kla‐modified proteins in DCM mice were used to analyze the features of protein lactylation (Kla) sites on each disease sample, including the number of sites and proteins detected on each sample, the correlation between disease and normal samples, the quantity of lactic acid proteins and Kla sites, and the subcellular localization of Kla proteins. The R packages ggplot2, ggpubr, and pheatmap were employed to create the aforementioned graphs.

The sites annotated to histones were found in the quantitative data of lactate, and the histone modifications on the disease and control samples were counted. Ggplot2 was used to plot, and the ggpubr package was applied to compute the differences between the two groups.

The standardized lactic acid quantitative data were used to screen the differential modification sites, and the ratio of the mean expression of the disease group to the control group was calculated as the fold change. The *t*‐test was used to calculate the significance of the *p* value of the difference between the two groups. The screening threshold was |*f* *o* *l* *d* *c* *h* *a* *n* *g* *e*| > 1.2 and *p* value < 0.05. The number of differentially expressed Kla sites and the number of corresponding proteins in the disease and control samples were analyzed, and the subcellular localization of differentially expressed Kla sites and proteins was also analyzed. The R packages ggplot2 and ggrepel were used to draw the volcanic map, and the pheatmap package was used to draw the expression heat map. The subcellular localization difference map was drawn using ggforce.

The corresponding proteins with high expressions of Kla sites on the samples of the disease and control groups were obtained, and the differential proteins were analyzed by Gene Ontology (GO) and Kyoto Encyclopedia of Genes and Genomes (KEGG) enrichment using the R package clusterProfiler.

The protein quantitative data were used to calculate the differences in proteins between the disease and the control samples, which were then combined with the differences in Kla sites. ggplot2 was used to draw a scatter plot of the differences in Kla sites and corresponding proteins, as well as calculate their correlation. In addition, the sites in each quadrant were analyzed, including metabolic genes, differential Kla sites, number of differential proteins, KEGG pathways, and GO functional annotation. Principal component analysis maps of DCM and control samples were displayed using quantitative lactate data and quantitative protein data, respectively, to evaluate the ability to distinguish different groups of samples based on the protein level compared with the lactate level.

Based on the differentially expressed Kla sites, Least Absolute Shrinkage and Selection Operator (LASSO) regression, logistic regression, random forest (RF), and support vector machine–recursive feature elimination (SVM‐RFE) algorithm were used to analyze the key lactic acid sites. The potential key lactic acid sites were obtained from the intersection of Kla sites screened by LASSO, logistic, SVM‐RFE, and RF. Subsequently, we used the R packages glmnet, randomForest, caret, and glm to perform LASSO regression, RF, SVM‐RFE, and logistic regression algorithm, respectively. Finally, the R package ggvenn was used to show the above‐mentioned intersection of Kla sites.

### 2.8. Statistical Analysis

All analyses were carried out using R Version 4.3.0. For the significance analysis, the Wilcoxon rank‐sum test was employed to compare differences between the two groups of samples.

## 3. Results

### 3.1. Characterization of Lactate Modification in DCM

**Figure 1:**
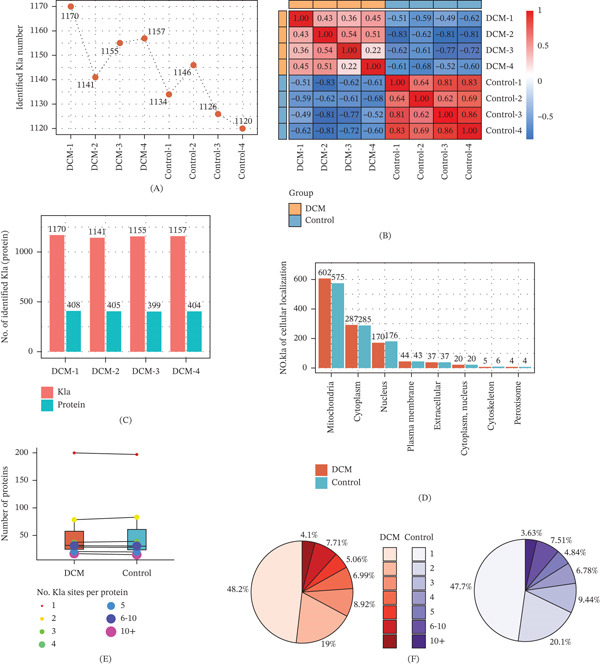
Characteristics of lactylation modification in the hearts of DCM mice. (A) Number of Kla sites detected in each sample. (B) Correlation among samples. (C) Number of Kla sites and corresponding proteins detected in the heart samples of DCM mice. (D) Subcellular localization of Kla‐modified proteins in the disease and control groups. (E) Average number of Kla per protein in the disease and control groups. (F) Proportion of proteins with different numbers of Kla sites in the DCM and control groups. DCM, diabetic cardiomyopathy.

To achieve a comprehensive molecular understanding of the Kla sites involved in DCM progression, proteome and lactylome analyses were performed on the heart tissues of mice from the control group and DCM group. The quantitative results of Kla modification sites in the two groups showed fewer Kla sites in the control group (1120–1134) than in the DCM group (1141–1170), indicating a significant increase in protein lactylation under disease conditions (Figure 1A). Correlation analysis showed robust intragroup correlations (*R* > 0.90) and significantly decreased intergroup correlations (*R* < 0.40), indicating strong consistency within either the DCM or the control group, with significant differences in Kla features observed between the two groups (Figure 1B). The ratio of Kla sites to protein (approximately three times) remained stable in all DCM samples (Figure 1C). Subcellular localization analysis indicated that the majority of Kla‐modified proteins were located in the mitochondria and cytoplasm. Moreover, in the DCM group, there was a slight increase in the nuclear localization of Kla‐modified proteins, suggesting that nuclear signal transduction or chromatin regulation may have changed (Figure 1D). Moreover, most of the Kla‐modified proteins had one or two or a small number of sites, and the proportion of proteins with more than six lactylation modification sites was rare (Figure 1D,E). Together, these findings highlight increased Kla in DCM, with distinct subcellular and protein‐specific enrichment patterns(Figure 1F).

### 3.2. Histone Lactate Modification in DCM

Histone lactate modification is a newly discovered epigenetic modification involved in glycolysis‐related cell functions that play crucial roles in numerous biological processes. Therefore, we analyzed Kla‐modified sites and intensities on histones in both the DCM and control groups.

**Figure 2:**
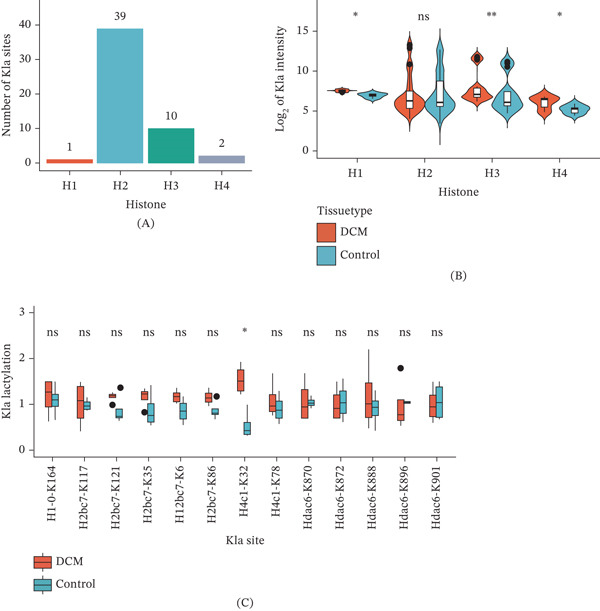
Overview of histone lactylation modification in DCM mice. (A) Number of Kla sites detected on different histones in the samples. (B) The Kla intensity on different histones in the disease and control groups. (C) Comparison of the Kla sites of lactylation on histones detected in the disease and control groups.

The results showed that H2 histones had the highest number of Kla‐modified sites (39), followed by H3 (10), H4 (2), and H1 (1) histones (Figure 2A). Kla intensities were significantly higher in the DCM group for H1, H3, and H4 histones (*p* < 0.05), whereas no significant difference was observed for H2 histones (Figure 2B). Although most annotated Kla sites showed no significant difference in lactylation levels between the two groups, the H4c1‐K32 site displayed significantly increased lactylation in DCM groups (*p* < 0.05, Figure 2C), indicating that H4c1‐K32 could serve as a potential key site for further exploration in understanding the epigenetic regulation of DCM. These findings suggest that Kla modifications occur across histones in a site‐ and type‐specific manner, potentially contributing to chromatin remodeling and gene expression regulation in DCM.

### 3.3. Differential Kla Modification Sites in DCM

**Figure 3:**
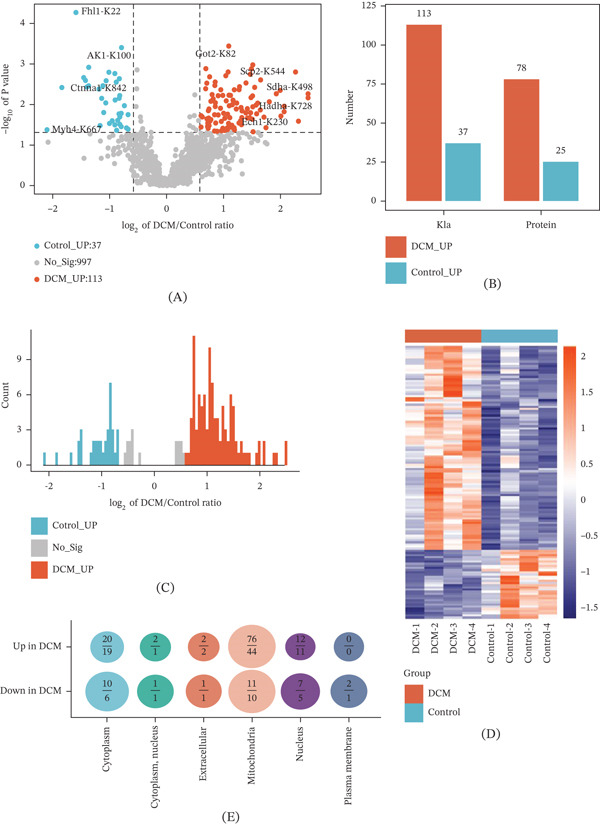
Sites with altered differential lactylation modification in the hearts of DCM mice. (A) Volcano plot of differential Kla sites between the disease and control groups. (B) Number of differential Kla sites and corresponding proteins in the disease and control groups. (C) Distribution of the ratio of Kla sites in the DCM group to those in the control group, with *p* < 0.05. (D) Clustering heat map of differential Kla. (E) Subcellular localization of Kla‐modified proteins corresponding to upregulated and downregulated differential Kla sites. DCM, diabetic cardiomyopathy.

Next, we screened the differential Kla sites between the two groups and uncovered that there were 113 significantly modified Kla sites corresponding to 78 modified proteins in the DCM group, whereas there were 37 significantly modified Kla sites corresponding to 25 modified proteins in the control group (Figure 3A,B). Compared with the upregulated Kla sites in the control group, Kla sites upregulated in the DCM group exhibited a wider range of log2 fold‐change values (Figure 3C). The heat map further illustrated distinct Kla expression patterns, with clear separation between the DCM and control samples (Figure 3D). The subcellular distribution results showed that the upregulated Kla‐modified proteins were mainly situated within the mitochondria and cytoplasm, whereas the downregulated Kla‐modified proteins were primarily enriched in cytoplasmic and nuclear compartments (Figure 3E). The above results indicate that Kla modification is elevated in DCM and exhibits significant, distinct site‐specific changes and subcellular enrichment patterns, which clearly differentiate disease and healthy tissues.

### 3.4. Enrichment Analyses of GO and KEGG for Proteins Related to Differential Kla Sites

**Figure 4:**
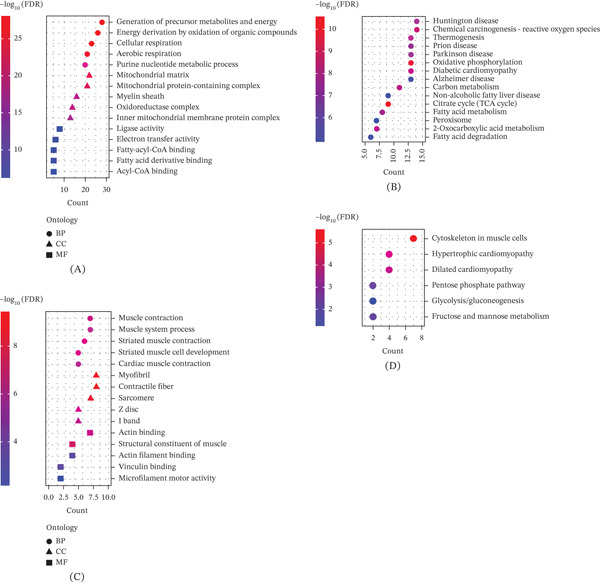
Functional study on the crosstalk of lactylation modification in DCM mice. (A) GO enrichment analysis of proteins corresponding to upregulated lactylation modification sites. (B) KEGG enrichment analysis of proteins corresponding to upregulated lactylation modification sites. (C) GO and KEGG enrichment analysis of proteins corresponding to downregulated lactylation modification sites. (D) KEGG enrichment analysis of proteins corresponding to downregulated lactylation modification sites. DCM, diabetic cardiomyopathy; GO, Gene Ontology; KEGG, Kyoto Encyclopedia of Genes and Genomes.

To understand the biological significance of differentially Kla‐modified proteins in DCM, functional enrichment analyses, focusing on their roles in cellular pathways and processes, were performed. Upregulated Kla‐modified proteins were enriched in energy metabolism–related biological processes, including the generation of precursor metabolites and energy, cellular respiration, and aerobic respiration, which aligned with the increased energy demands of the diseased heart. Cellular component analysis revealed significant enrichment in mitochondrial structures, such as the mitochondrial matrix and the oxidoreductase complex, whereas molecular function analysis highlighted fatty acyl‐CoA binding and electron transfer activity (Figure 4A). KEGG pathway analysis further confirmed the involvement of these proteins in key metabolic pathways, including oxidative phosphorylation, the TCA cycle, and fatty acid degradation (Figure 4B), emphasizing the centrality of mitochondrial and metabolic processes in DCM pathogenesis.

In contrast, downregulated Kla‐modified proteins were associated with the structural and functional aspects of cardiac muscle. GO analysis revealed enrichment in processes such as muscle contraction, the muscle system, and cardiac muscle contraction, and cellular component analysis showed significant associations with sarcomeric and cytoskeletal structures, including I band and actin filament, whereas molecular functions such as the structural constituent of muscle and actin binding were also prominent (Figure 4C). KEGG pathway analysis identified key cardiac‐related pathways, including dilated cardiomyopathy and hypertrophic cardiomyopathy, alongside metabolic pathways like glycolysis/gluconeogenesis (Figure 4D).

Overall, these results suggest that upregulated Kla proteins in DCM contribute to increased metabolic activity, particularly mitochondrial energy production, whereas downregulated Kla proteins are involved in the loss of structural and functional integrity of cardiac muscle. This dual alteration in Kla‐modified proteins reflects the interplay between metabolic and contractile dysfunctions in DCM.

### 3.5. Correlation Between Lactylome and Proteome in DCM

**Figure 5:**
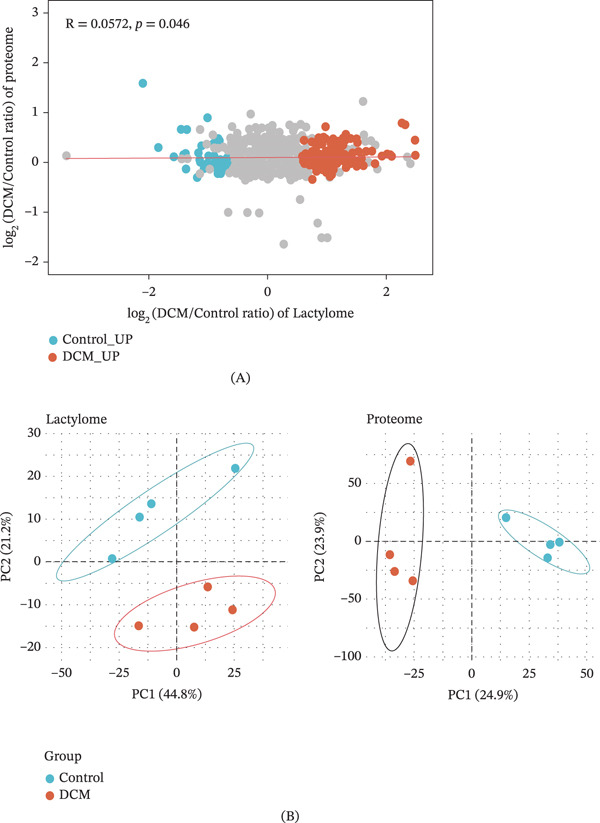
The level of lactylated proteins and Kla sites in all samples. (A) Correlation between lactylated protein level and the Kla site lactylated level of all samples, with different colors indicating different lactylated sites. (B) The ability of the Kla site lactate level and protein level to distinguish between disease and control.

To gauge the interaction between lactylation levels and protein abundance in DCM, their correlation was examined, and PCA was used to evaluate their capacity to distinguish disease from control samples. A subtle yet statistically notable positive correlation (*R* = 0.0572, *p* = 0.046) was detected between the log2 fold changes in Kla‐modified sites (lactylome) and their corresponding protein levels (proteome) (Figure 5A). PCA further revealed distinct clustering of DCM and control samples in both the lactylome and proteome datasets (Figure 5B). The lactylome demonstrated a greater degree of variance, which was explained by the first principal component (PC1: 44.8%) compared with the proteome (PC1: 24.9%), highlighting its stronger ability to separate DCM from control samples. These results suggest that Kla modifications are partly associated with protein abundance; however, they also exhibit unique patterns reflective of disease states, thus supporting their potential role as disease‐specific biomarkers in DCM.

**Figure 6:**
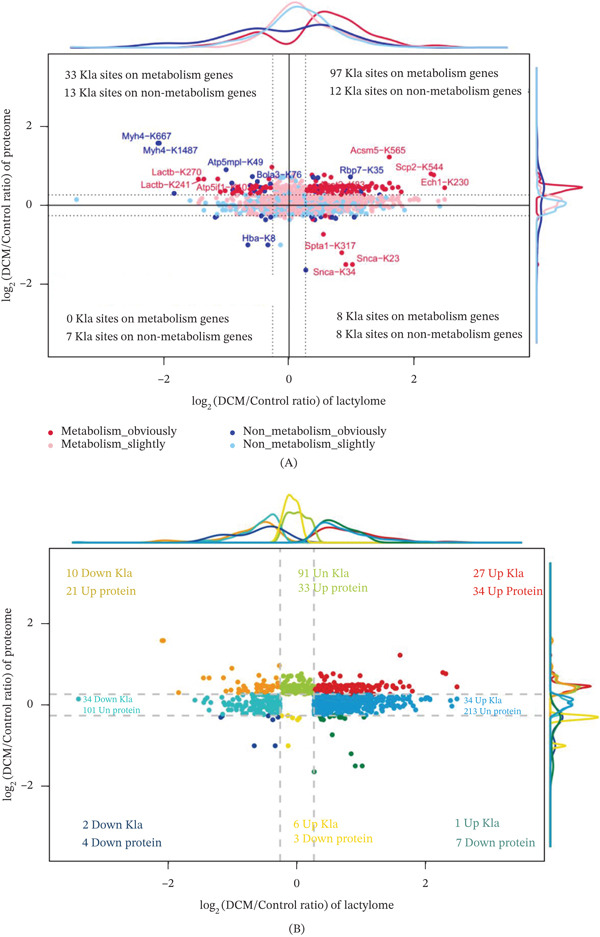
Characteristics of lactylated proteins and Kla site modification levels. (A) With a fold change of 1.2 as the difference level, the number of Kla sites on metabolic and nonmetabolic genes under different conditions was shown. (B) With a fold change of 1.2 as the difference level, the number of lactylated proteins and Kla sites under different conditions was calculated.

Quadrant analysis revealed distinct patterns of lactylation changes in metabolic and nonmetabolic genes. Kla sites on metabolic genes were predominantly upregulated in DCM, especially in Quadrant I, which represents both increased Kla modifications and protein abundance. Key Kla‐modified sites include Got2‐K82, Sdha‐K498, and Ech1‐K230, highlighting the enhanced lactylation of proteins involved in metabolic pathways, whereas nonmetabolic genes showed a more balanced distribution, with some downregulated Kla sites, particularly in Quadrants II and III, where protein and Kla modifications showed discordant changes (Figure 6A). The distribution of Kla protein pairs across all quadrants further supports this trend. Quadrant I contained the majority of Kla sites (251), corresponding to 109 upregulated proteins, thus suggesting a strong coordination between Kla modification and protein abundance in metabolic pathways. Conversely, Quadrants II and IV, where Kla and protein changes were decoupled, had relatively few pairs, underscoring the limited discordance in these patterns (Figure 6B). This pattern suggests that lactylation may play a role in enhancing metabolic activity, which is a key feature of DCM.

### 3.6. Functional Enrichment of Quadrant‐Specific Kla–Protein Interactions

**Figure 7:**
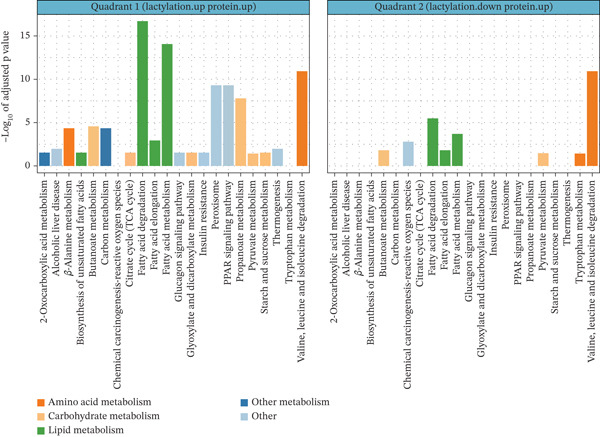
KEGG enrichment analysis results of Kla‐modified proteins in each quadrant. KEGG, Kyoto Encyclopedia of Genes and Genomes.

**Figure 8:**
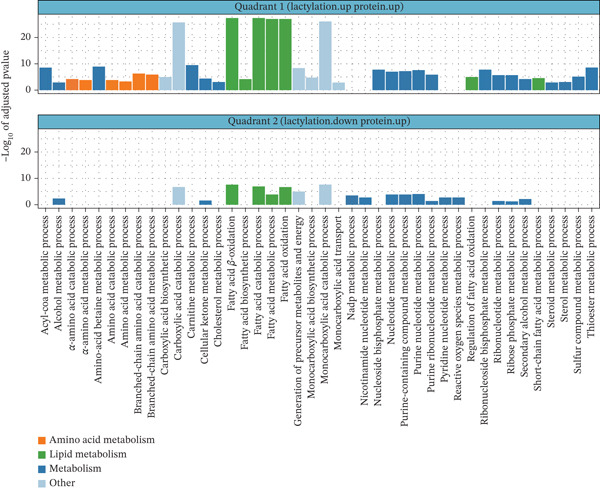
GO enrichment analysis results of Kla‐modified proteins in each quadrant. GO, Gene Ontology.

To further understand the metabolic implications of Kla modifications, KEGG and GO enrichment analyses were performed on proteins from Quadrants I (upregulated Kla and protein) and II (upregulated Kla and downregulated protein), focusing on their roles in disease‐associated pathways. In Quadrant I, KEGG enrichment showed pathways associated with energy production, including the TCA cycle, glycolysis/gluconeogenesis, and fatty acid degradation (Figure 7, left panel). GO enrichment analysis highlighted biological processes such as the generation of precursor metabolites and energy, as well as fatty acid metabolic processes (Figure 8, top panel). These pathways indicate that Kla modifications enhance the activity of metabolic proteins, which are predominantly involved in lipid and amino acid metabolism, further linking lactylation to energy metabolism and nutrient processing. Proteins in this quadrant were predominantly involved in lipid and amino acid metabolism, further linking lactylation to energy metabolism and nutrient processing.

In Quadrant II, KEGG analysis revealed a broader spectrum of pathways, with notable enrichment in the biosynthesis of amino acids, glyoxylate and dicarboxylate metabolism, and PPAR signaling pathways (Figure 7, right panel). GO enrichment analysis highlighted diverse processes, including fatty acid metabolism and amino acid catabolism, but with lower statistical significance compared with Quadrant I (Figure 8, bottom panel). This suggests that while Kla may compensate for reduced protein abundance, its role is less pronounced in driving major metabolic shifts.

Together, Kla modifications are most strongly associated with upregulated proteins involved in energy production and amino acid metabolism, particularly in Quadrant I. In contrast, Kla modifications may act as an adaptive mechanism to modulate metabolic and biosynthetic pathways under the conditions of reduced protein abundance in DCM.

### 3.7. Identification of Key Kla Sites in DCM Using Machine Learning

**Figure 9:**
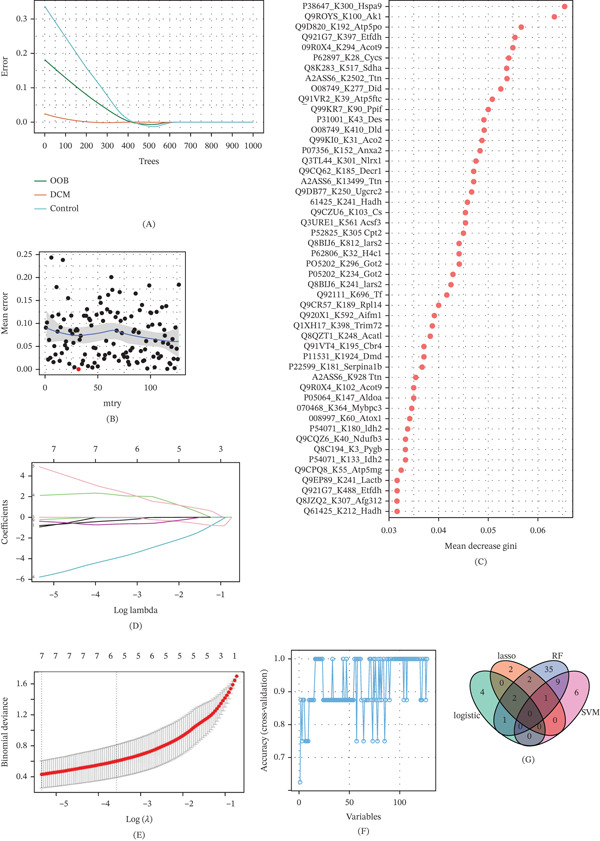
Multiple machine learning methods used to identify key lactylation sites in DCM. Four complementary algorithms were employed to ensure robustness when screening variables: LASSO and logistic regression were used to simplify the data by removing redundant factors, identifying linear contributions, and reducing data redundancy. RF and SVM‐RFE were applied to capture complex, nonlinear patterns via decision‐tree ensembles and pinpoint the most discriminative feature subset through iterative elimination, respectively. (A) Error rate fluctuation curves represent increases in the number of trees in the RF algorithm. (B) Mean error rate of the RF algorithm as a function of the mtry parameter. (C) Selection of the Top 50 sites based on the Gini coefficient ranking in the RF. (D) LASSO coefficient profiles as a function of the log(lambda) sequence. (E) Tenfold cross‐validation error curves for LASSO regression. (F) Cross‐validation plot for the SVM method. (G) Intersection of variables selected by the four machine learning algorithms. DCM, diabetic cardiomyopathy; LASSO, Least Absolute Shrinkage and Selection Operator; RF, random forest; SVM‐RFE, support vector machine–recursive feature elimination.

To identify critical Kla‐modified sites linked to DCM, four machine learning approaches were utilized, including RF, LASSO regression, SVM‐RFE, and logistic regression. The RF model achieved stability at 600 decision trees, where the error rate plateaued (Figure 9A). Optimal performance was observed at mtry = 32, which minimized mean error (Figure 9B). Ranking features by Gini importance revealed the top Kla sites, including P38647_K30_Hspa1, Q80U76_K36_Rp2, and O88943_K3_Maf1 (Figure 9C). LASSO regression identified seven significant Kla sites after selecting the optimal lambda through cross‐validation (Figure 9D,E). SVM‐RFE achieved the highest classification accuracy when 16 variables were retained, further narrowing the list of candidate sites (Figure 9F). Logistic regression added additional features, partially overlapping with the results from other methods. By integrating the outputs from all four methods, we identified five consensus Kla sites—A2ASS6_K928_Ttn, A2ASS6_K13499_Ttn, Q61425_K212_Hadh, Q8K2B3_K517_Sdha, and Q9R0Y5_K100_Ak1—that were consistently selected by at least two approaches (Figure 9G). These sites are potential key lactylation targets in DCM, warranting further experimental validation.

## 4. Discussion

DCM is a unique complication of diabetes that increases the risk of heart failure and mortality. The number of diabetic patients is growing at an alarming rate worldwide due to changes in lifestyle [20]. In addition to controlling metabolic disorders, the current clinical treatment strategies for DCM focus on antifibrosis agents, anti‐inflammatory agents, and antioxidants [21]. Despite an increase in the approval of DCM, effective therapeutic strategies remain limited, owing to the complex etiology and pathogenesis of DCM [22]. In addition, there is a lack of unified criteria for the diagnosis of DCM [23]. Therefore, it is crucial to discover the potential pathogenesis of DCM, leading to more effective treatments. For the first time, this study comprehensively profiles the lactylome and proteome in cardiac tissues of db/m mice and db/db mice, which may help to elucidate the pathogenesis of DCM.

Thanks to the advancement of high‐resolution mass spectrometry technology, a variety of acylation modifications based on intermediate metabolites derived from substrates have been discovered. Protein lactylation is a posttranslational modification discovered by Zhao et al. in 2019 [14]. More recently, the interaction between metabolic reprogramming and protein modification has been well demonstrated [24–25]. Given that DCM is a metabolic disorder, we speculate that protein lactylation may be involved in the pathogenesis of DCM. In this study, the objective was to clarify the mechanism of DCM by investigating the protein lactylation landscape. Herein, we discovered that there were more Kla sites in the DCM group compared with the control group. Subsequent subcellular localization analysis indicated that Kla‐modified proteins were predominantly located in the mitochondria and cytoplasm in both the DCM and control groups. The main pathways of glucose metabolism include the glycolysis pathway, the tricarboxylic acid cycle, and oxidative phosphorylation, which mainly occur in the cytoplasm and mitochondria. Recently, a rapidly increasing number of studies have attempted to treat DCM by improving glucose metabolism [26–28]. For instance, Nrf2 improves glucose metabolism by directly targeting AMPK/Sirt1/PGC‐1a through upstream genes sestrin2 and LKB1 in DCM mice, and indirectly activates AKT/GSK‐3*β*/HK‐II activity through AMPK‐mediated p70S6K inhibition, thereby exerting myocardial protection. Based on this, we speculate that these Kla‐modified proteins could play a role in the development of DCM.

Recently, histone modification has attracted increasingly more attention from researchers in discussing the pathogenesis and treatment of DCM [29–30]. Histone lactate modification is a new epigenetic modification that involves cell functions related to glucose metabolism. It holds a pivotal position in numerous biological processes [31–32]. In this study, protein lactate modification in both the DCM and control groups mainly occurred on the H2 histone. Additionally, the intensity of Kla on different histone proteins differed between the DCM group and the control group. Of note, we also discovered that only the lactate modification that occurred at the H4c1‐K32 site was much higher in the DCM group than in the control group. We hypothesize that the lactate modification occurring at the H4c1‐K32 site could potentially act as a biomarker for DCM diagnosis. Therefore, the underlying mechanisms of H4c1‐K32 deserve further study. Next, we found that there were differences in the significantly modified Kla sites between the DCM group and the control group, with 113 significantly modified Kla sites corresponding to 78 modified proteins in the DCM group, whereas 37 significantly modified Kla sites corresponded to 25 modified proteins in the control group. In addition, we also found that the DCM group was mainly upregulated at Kla modification sites, and the proteins corresponding to these sites were not only localized in the mitochondria and cytoplasm, but also distributed in the nucleus to a certain extent. Therefore, in‐depth analysis of these lactate modification sites in the future will lead to understanding the mechanisms underlying DCM pathogenesis.

GO and KEGG enrichment analysis was performed on the corresponding proteins of differential Kla sites to study the functions of proteins involved in lactate modification in DCM. We discovered that lactate‐modified proteins, whether upregulated or downregulated, participated in biological processes and pathways associated with glucose metabolism and DCM, such as phosphorylation, citrate cycle (TCA cycle), oxidation of organic compounds, glycolysis/gluconeogenesis, and DCM. Thus, we speculate that these Kla‐modified proteins might be engaged in regulating DCA progression by mediating these biological processes and pathways.

Machine learning approaches are increasingly applied in epigenetic research. These methods can identify key epigenetic features, interpret the association between phenotype and histone or gene modifications, and accelerate target screening, which may ultimately achieve the development of precision medicine [33–34]. Based on the selected differential Kla sites, four machine learning methods—LASSO regression, logistic regression, RF, and SVM‐RFE—were used to further screen the key Kla sites. Ultimately, five Kla sites were obtained as candidate sites, namely A2ASS6_K928_Ttn, A2ASS6_K13499_Ttn, Q61425_K212_Hadh, Q8K2B3_K517_Sdha, and Q9R0Y5_K100_Ak1. Therefore, we speculate that these five candidate sites could be pivotal in the development of DCM. However, there are limitations in the use of animal models in this study and potential considerations in their translation to clinical settings. This study employed a db/db mouse model, which was broadly applied in DCM due to its characteristic hyperglycemia and metabolic disturbances. However, animal models cannot reflect the mechanism of the heterogeneous pathogenesis of human DCM completely, including complex interactions among metabolic, genetic, and environmental factors [1, 34]. Therefore, caution should be taken when applying this finding in clinical settings. To further study the function of the five candidate sites in DCM, a large number of samples and a series of experiments are needed.

Protein lactylation may interact with several established pathogenic mechanisms. Chronic hyperglycemia promotes oxidative stress, mitochondrial dysfunction, and the accumulation of advanced glycation end products, which can cause myocardial injury [17, 35]. Under diabetic conditions, lactate production may enhance the lactylation of metabolic enzymes via glycolytic metabolism and therefore influence mitochondrial function and redox balance. Both lactylation and glycation target lysine residues on proteins, which indicates that these metabolite‐driven modifications may interact under hyperglycemic conditions. This type of interaction may represent metabolic reprogramming with epigenetic and proteomic alterations in DCM.

In this study, we performed integrated proteome and lactylation analyses of cardiac tissues from control and DCM mice. This approach enabled us to systematically characterize the distribution and abundance of Kla sites. In addition, we found that modification of H4c1‐K32 sites in the DCM group was significantly higher compared with the control group. Proteins with Kla modification participated in biological processes and pathways related to glucose metabolism and DCM. Finally, four machine learning methods were combined to identify and select five candidate Kla sites, namely A2ASS6_K928_Ttn, A2ASS6_K13499_Ttn, Q61425_K212_Hadh, Q8K2B3_K517_Sdha, and Q9R0Y5_K100_Ak1. Our findings suggest that protein lactate modification could be a promising potential treatment approach for DCM. Future studies of differential proteins and their modification sites are needed to develop new and effective therapeutic targets for treating DCM.

## Author Contributions

Di Ma: methodology and writing—original draft; Wenjie Cai: data curation and methodology; Hui Yuan: software and writing—original draft; Meixin Shi: data curation and visualization; Ye Jin: data curation and visualization; Yifeng Cui: methodology; Peng Liu: resources; Xi Liu: project administration and conceptualization; Can Wei: project administration, conceptualization, resources, data curation, and supervision. Di Ma, Wenjie Cai, and Hui Yuan have contributed to the work equally and should be regarded as cofirst authors.

## Funding

This study was supported by the National Natural Science Foundation of China (No. 82170268) and the Key Research and Development Project of Ordos (No. YF20240051).

## Ethics Statement

The mice were treated following the Guide for the Care and Use of Laboratory Animals, as adopted and promulgated by the United States NIH. All treatment protocols received approval from the Institutional Animal Care and Use Committee at the Experimental Animal Center of Harbin Medical University (Harbin, Heilongjiang, China).

## Conflicts of Interest

The authors declare no conflicts of interest.

## Data Availability

Proteomics data have been deposited in the ProteomeXchange Consortium via the PRIDE repository under Accession Number PXD067368. Processed data will be made available on request.
